# Optimal timing of antimicrobial prophylaxis before surgery: a review of recent evidence

**DOI:** 10.17179/excli2021-4472

**Published:** 2021-12-10

**Authors:** Saarat Sathoo, Vimal Thomas, Amudhan Kannan, Divya Bajaj, Anirudh Abu Srinivasan

**Affiliations:** 1 Jawaharlal Institute of Postgraduate Medical Education and Research (JIPMER), Puducherry, India; 2 Tbilisi State Medical University, Tbilisi, Georgia

## ⁯⁯⁯⁯

***Dear Editor,***

**Figure 1:**
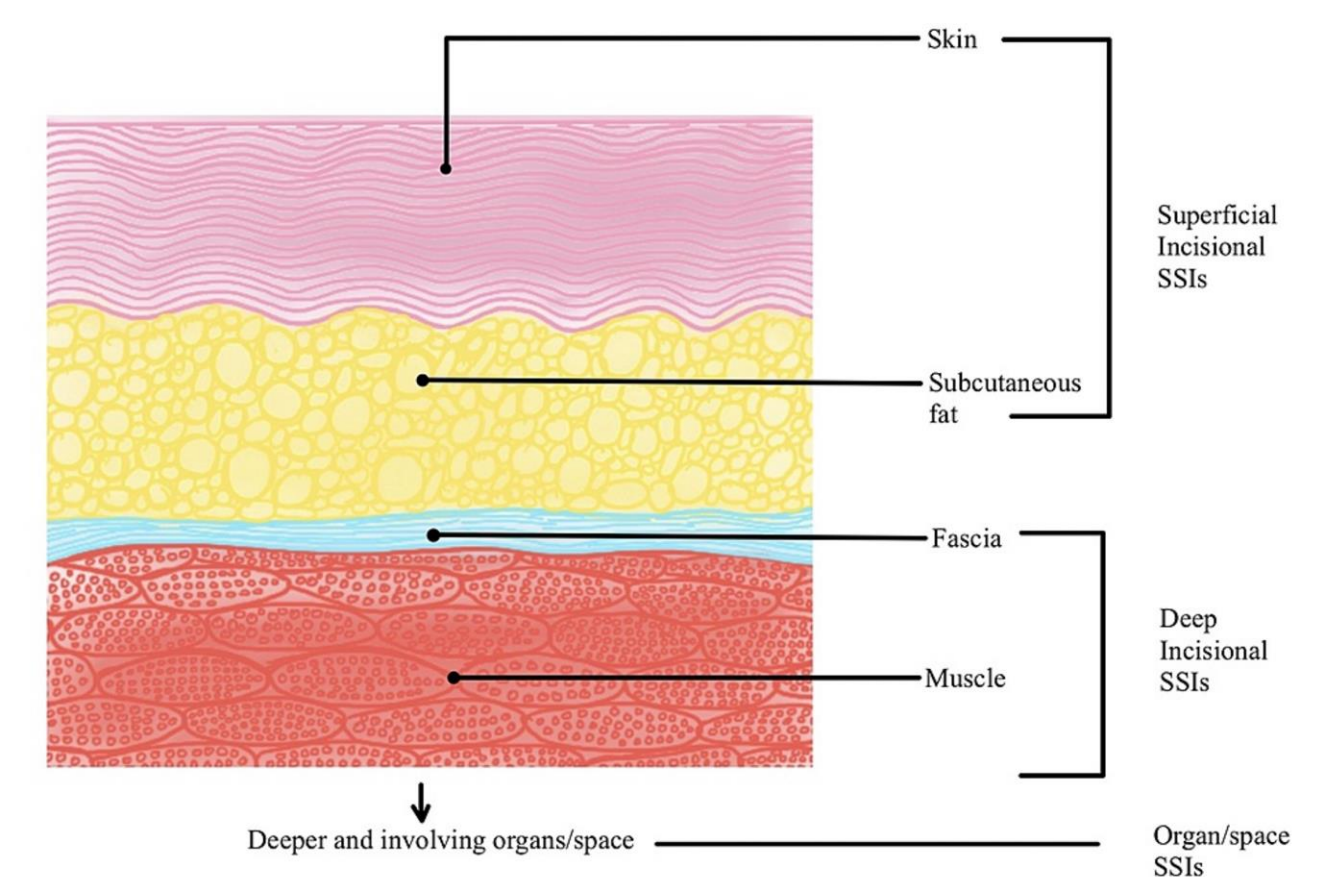
Classification of Surgical Site Infections (SSIs). Picture courtesy: Kannan et al. (2021)

According to the centers for disease control and prevention (CDC), surgical site infections (SSIs) are the infections that occur after the surgery, either involving the skin or involving the deep tissues (Berríos-Torres et al., 2017[1]). The CDC divides surgical site infections into three groups: superficial incisional SSI, deep incisional SSI, and organ/space SSI (Figure 1(Fig. 1); Reference in Figure 1: Kannan et al., 2021[4]). SSIs are more commonly seen in patients with more comorbidities and patients undergoing emergency surgeries. In addition to increased morbidity and mortality following surgery, SSI contributes to increased healthcare expenses and worsens the patient's quality of life (Kannan et al., 2021[4]). Therefore, it is crucial to prevent SSIs, thus avoiding the establishment of complications. The best way to tackle this problem is to provide the patients with antibiotic prophylaxis before surgery.

The World Health Organization (WHO, 2018[6]) recommends preoperative antibiotic administration 120 minutes before the skin incision, however, keeping in mind the half-life of the antibiotic being administered. The CDC recommends using antimicrobial prophylaxis at a time before skin incision such that the antibiotic concentration reaches the minimum bactericidal concentration at the time of skin incision. There is no subsequent refinement of the timing of antibiotic administration which can be made. The CDC also recommends antibiotic prophylaxis at the time of skin incision for cesarean sections.

The appropriate antibiotic and its dose are chosen depending on the microbial flora, its complications, and patient risk factors. Kannan et al. (2021[4]) had done a review to determine the sufficient number of prophylactic antimicrobial doses that are efficacious in controlling the SSIs following GI oncological surgeries. The authors concluded that single-dose antimicrobial prophylaxis had the same efficacy as the multiple-dose antimicrobial regimen in controlling SSIs in esophageal, gastric, and colorectal surgeries. The advantages of a single-dose regimen told in the review include less chance of emergence of resistance, less chance for allergies or toxicity, and less cost. 

Recently we read with great interest the article by de Jonge et al. (2021[2]). We congratulate the authors on the great efforts they have put into the study. This is a cohort study. The authors had included 3,001 patients who received antimicrobial prophylaxis for general, orthopedic, or gynecologic surgery. The occurrence of SSI was defined according to the CDC guidelines. Of the 3,001 patients, 2612 patients received antibiotic prophylaxis within 60 minutes before incision, of whom 1550 patients received SAP between 0 and 30 minutes and 1062 patients received between 30 and 60 minutes before incision. The remaining 389 patients received prophylaxis 60-120 minutes before the incision. On analysis, the authors found out that the incidence of superficial and deep SSIs was lower in the 60-120 minutes cohort. But the authors could not conclude its superiority due to the lower number of observations in the 60-120 minutes cohort. 

De Jonge et al. (2017[3]) had done a systematic review to assess the effect of timing of preoperative surgical antibiotic prophylaxis on SSI and compared the different timing intervals. The authors did not identify any significant difference in the incidence of SSI within this 120-minute time frame prior to incision. Weber et al. (2017[5]) did a randomized controlled trial (RCT) to determine the optimal timing of surgical antimicrobial prophylaxis. A total of 5580 patients were randomly assigned to the early group, i.e., 30-75 min before the incision (2798 patients), or the late group, i.e., 0-30 min before the incision (2782 patients). Out of this, 5175 patients were analyzed. The authors did not find any significant difference between the two groups and concluded that any narrowing of the 60-min window for administering antibiotics with a short half-life is not beneficial.

Therefore, the results of de Jonge et al. (2021[2]) cohort study about the difference in timing of antibiotic prophylaxis within 120 minutes of the incision should be interpreted cautiously, and this aspect should be studied further. We recommend systematic reviews and meta-analyses on this topic to get a consolidated conclusion about the optimal timing of surgical antimicrobial prophylaxis.

## Conflict of interest

The authors declare no conflict of interest.
